# A multi-class brain tumor grading system based on histopathological images using a hybrid YOLO and RESNET networks

**DOI:** 10.1038/s41598-024-54864-6

**Published:** 2024-02-26

**Authors:** Naira Elazab, Wael A. Gab-Allah, Mohammed Elmogy

**Affiliations:** Information Technology Department, Faculty of Computers and Information, Mansoura University, Mansoura, 35516 Egypt

**Keywords:** Brain tumor, Histopathology images, Brain tumer grades diagnosis, Deep learningm YOLOv5, Resnet50, Computer science, Information technology

## Abstract

Gliomas are primary brain tumors caused by glial cells. These cancers’ classification and grading are crucial for prognosis and treatment planning. Deep learning (DL) can potentially improve the digital pathology investigation of brain tumors. In this paper, we developed a technique for visualizing a predictive tumor grading model on histopathology pictures to help guide doctors by emphasizing characteristics and heterogeneity in forecasts. The proposed technique is a hybrid model based on YOLOv5 and ResNet50. The function of YOLOv5 is to localize and classify the tumor in large histopathological whole slide images (WSIs). The suggested technique incorporates ResNet into the feature extraction of the YOLOv5 framework, and the detection results show that our hybrid network is effective for identifying brain tumors from histopathological images. Next, we estimate the glioma grades using the extreme gradient boosting classifier. The high-dimensional characteristics and nonlinear interactions present in histopathology images are well-handled by this classifier. DL techniques have been used in previous computer-aided diagnosis systems for brain tumor diagnosis. However, by combining the YOLOv5 and ResNet50 architectures into a hybrid model specifically designed for accurate tumor localization and predictive grading within histopathological WSIs, our study presents a new approach that advances the field. By utilizing the advantages of both models, this creative integration goes beyond traditional techniques to produce improved tumor localization accuracy and thorough feature extraction. Additionally, our method ensures stable training dynamics and strong model performance by integrating ResNet50 into the YOLOv5 framework, addressing concerns about gradient explosion. The proposed technique is tested using the cancer genome atlas dataset. During the experiments, our model outperforms the other standard ways on the same dataset. Our results indicate that the proposed hybrid model substantially impacts tumor subtype discrimination between low-grade glioma (LGG) II and LGG III. With 97.2% of accuracy, 97.8% of precision, 98.6% of sensitivity, and the Dice similarity coefficient of 97%, the proposed model performs well in classifying four grades. These results outperform current approaches for identifying LGG from high-grade glioma and provide competitive performance in classifying four categories of glioma in the literature.

## Introduction

A histopathological examination is required for cancer diagnosis. The classification of brain cancer patients is primarily based on histological findings that appropriately identify the kind of malignancy^1^. For a proper diagnosis and treatment strategy, histopathological examination of glioma tumor tissue is necessary. The use of histological imaging enables a thorough analysis of the tumor tissue, which is essential for determining the kind, grade, and extent of the tumor as well as tracking the response to treatment. Pathologists can precisely evaluate a patient’s status by examining tissue slice photographs from actual patients. Computer vision-based automatic histopathology diagnosis can assist pathologists in reducing their burden^2^.

In recent years, histopathology has become an important tumor detection and prognosis tool. Although such an idea is not a product of modernity, the scarcity of resources has long stifled its development. Therefore, recent technological advancements have greatly facilitated the widespread use of histopathological images in various applications. In contrast to traditional glass slides, the innovative whole slide images (WSI) are numerical reproductions of stained specimen materials^3^. Due to the ease of data sharing and archiving that these images provide, they are also significantly impacting the procedure of making a pathology diagnosis. The WSI analysis gives doctors a complete understanding of the data content and makes it possible to diagnose tumors and cancer subtypes accurately^4^. The segmentation and classification of WSI have been addressed using a variety of methodologies during the past few years. Most of these experiments concentrated on learning superficial aspects, such as texture and pattern recognition, as described in^5^, grey level co-occurrence matrix or local binary pattern^6^.

**Figure 1:**
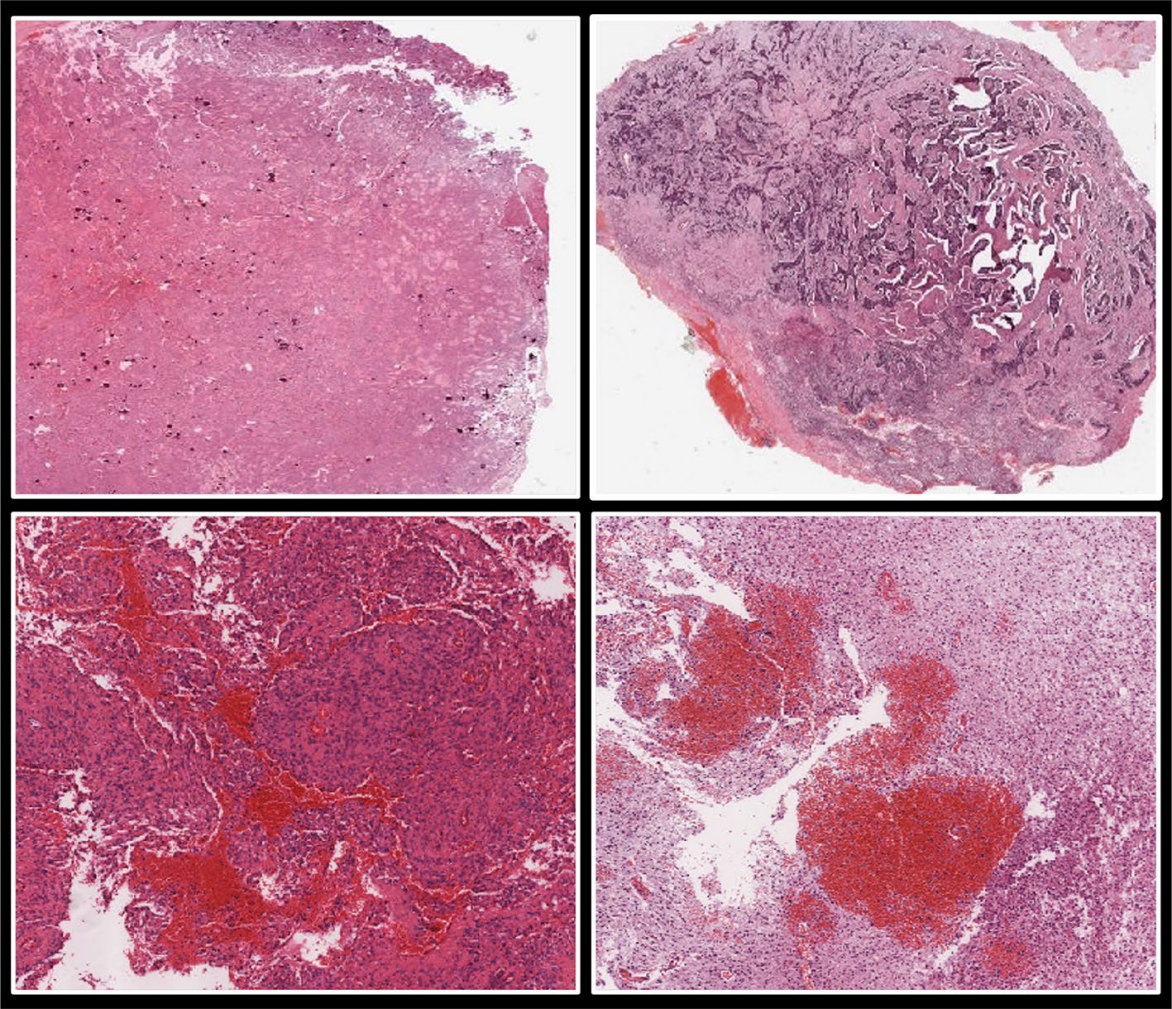
Samples of TCGA histopathological images.

However, each tumor contains a variety of textures, shapes, and color distributions. As a result, while dealing with the issues posed by current WSI, the previously discussed solutions are frequently constrained. Today histopathology images are quite big, containing billions of pixels. These slides typically present high-level, complicated clinical aspects. Furthermore, most of the time, they only represent a subset of the accessible annotated regions. Manually marking each WSI is time-consuming and requires significant effort and dedication^7^. Figure 1 shows different samples of glioma histopathological images.

The unique characteristics of histopathology pictures have sparked efforts to develop new automated image analysis methods. This circumstance can reduce pathologists’ effort, synchronize clinical applications, and reduce processing and handling time. Indeed, artificial intelligence (AI) models have gradually progressed from expert systems to classical machine learning and, finally, deep learning (DL)^8^. In other words, in the traditional hand-crafted systems, the data analysis activities relied heavily on expert knowledge to determine the relevant properties. However, the newest generation of DL networks can accurately and simply learn characteristics from the data itself^9,10^. Without a doubt, the development of highly effective computational resources has shifted the emphasis away from DL models in various applications for medical image processing. A significant part of current research in histopathology is focused on developing new DL models that will allow accurate analysis and information extraction from WSI^11^.

Effective diagnosis and treatment of brain tumors depend on careful analysis and information extraction from WSI. Brain tumors can be life-threatening, and early detection and classification are crucial for prompt and efficient patient treatment^12^. This is why computer-aided diagnostic (CAD) systems for brain tumor classification have been created. These technologies allow radiologists to visualize and categorize different forms of tumors. They can be beneficial when pathologists need a more thorough visual examination or are unsure of the tumor’s nature^13^. Researchers working on image processing and computer vision are concentrating on developing precise and effective algorithms for automatic tumor segmentation, classification, and identification. Their aim is to give clinicians reliable resources for making accurate diagnoses and giving patients prompt, efficient treatment^14,15^.

CAD is a routine clinical detection method widely used in many screening sites and hospitals. It has grown into an essential diagnostic imaging research field. Because of recent advancements in the digital preservation of digitized histological research, histopathological tissues are utilized with CAD systems to improve disease categorization^16^. Reviewing several slides and noticing inter- and intra-differences is exceedingly laborious and time-consuming for a pathologist. The traditional evaluation method employing histopathology pictures needs to be supported appropriately because many activities would be subject to these analysis-related problems. Workload should also be reduced to allow pathologists to focus on suspected cases that are difficult to diagnose. This can be accomplished by removing the innocuous examples. When diagnosing and comprehending a particular disease’s causes, quantitative analysis of pathology images is essential^17^.

Convolutional neural network (CNN) models have shown to be quite successful in complicated object recognition and classification tasks in recent years. Their main advantage is their capacity to extract sturdy characteristics resistant to varying degrees of distortion and light. DL has not yet addressed some histopathology issues, despite the fact that DL has shown remarkable results in several disciplines for identifying scenes and classifying objects. First, the histopathology field is constrained by the numerous data with labels needed for deep CNN training. Second, deep networks struggle to generalize successfully for new data after training on little data, making them susceptible to “overfitting.” Third, many computation resources are needed for deep CNN training, which often necessitates a lot of specialists’ prolonged devotion^18^.

The pathologist’s manual brain cancer diagnosis is time-consuming, exhausting, and burdensome. Few studies have been conducted to determine the grade of a brain tumor^19^. On the other hand, established automated systems that aid pathologists in cancer grade categorization depend on human-crafted feature tools that burden developers^20^. In addition to the richness and complexity of the retrieved features, these methods are sensitive to noise, contrast, and staining in digital histology images. The small variances between features make it difficult to easily extract the required features. Deeply tuned CNNs typically outperform fully trained CNNs when working with a limited training dataset, nonetheless.

Considering these issues, we present a new CAD system based on transfer learning that uses histopathological images to reliably diagnose healthy and glioma grades. The system begins with some preliminary activities. Normalization and various transformation methods were applied to standardize image sizes, maximize the restricted datasets, and avoid overfitting. We offer a new hybrid CNN model during the modeling phase. Without requiring manual feature extraction, or segmentation, the suggested model can identify various glioma grades. Based on transfer learning, we created a hybrid model that combines the YOLOv5^21^ and ResNet50 models^22^. We achieved a comprehensive model by combining the strong feature extraction and classification capabilities of ResNet-50 with the object detection prowess of YOLOv5. Together, these synergistic effects enabled the model to improve overall diagnostic accuracy by not only correctly identifying regions of glioma tumors but also picking up on fine features that are essential for accurate classification.

YOLOv5’s localization capabilities were enhanced by ResNet-50’s mastery of feature extraction and classification. Because of its depth and feature representation capabilities, the localized tumor regions could be analyzed more deeply, which made it easier to classify the data accurately based on patch-level information that was extracted. The generated feature vectors are fed into the extreme gradient boosting (XGBoost) classifier once features from the hybrid model have been extracted. For classification applications requiring high-dimensional features, such as those obtained from histopathology images, XGBoost is a potent and well-known gradient-boosting classifier. We compared the proposed system to other models and calculated important metrics for its performance. A few advantages of the two-hybrid models are their decreased complexity, increased robustness, and greater generalization and inference capabilities.

The major contributions of our proposed system are summarized in the following points:

- We developed an enhanced model for grading different types of brain tumors based on pathological images.
- BY using transfer learning, we created a hybrid model that integrates the customized YOLOv5 and the ResNet50 models for feature detection.
- The proposed model is effective because it combines depth-wise various scales of YOLO with identity mapping of ResNet50, which improves model performance by reducing the vanishing gradient issue and enhancing backward gradient flow in the network.
- We modify the YOLOv5 model’s backbone to enhance the feature extraction process and reduce the number of parameters to decrease computationally.
- The performance of our framework was compared to that of other state-of-the-art methods, and it performed the best.

This paper is structured as follows for the remaining sections: “Related work” section explains the related work of classifying multi-grade brain tumors. With an emphasis on augmentation, and fine-tuning of the used architecture, “The proposed framework” section presents the proposed methodology. “Experimental results” section discusses the conducted experiments. The conclusion of the paper is provided in “Conclusion and future work” section.

## Related work

Numerous researchers have used computer algorithms for histology image processing, and the approaches used can be divided into two categories: hand-crafted and DL techniques. The hand-crafted techniques extract features from slide images that professional pathologists would recognize. In contrast, the second category used DL techniques to automatically extract the features from the processed images. Due to their capacity to learn pertinent features directly from the raw data, Dl models, particularly CNNs, have been used more frequently in histopathology image analysis. In contrast, conventional ML systems rely on manually created characteristics. Deep learning has demonstrated considerable potential in capturing complicated patterns and variances in histopathology images when compared to conventional approaches, making it an important tool for developing the field.

Many studies use DL to analyze brain tumor histopathology images. According to cellular level features collected from hematoxylin and eosin (H &E) histopathology images of brain tumours, Sumi et al.^23^ suggested spatial fusion network design can categorize four different types of brain tissues. Before training the InceptionResNetv2 (INRV2) architecture to predict probabilistic features of various tumor cancers for local patches, the model extracts patches from each image and applies augmentation to them. A deep spatial fusion network is put into place in the second step to discover the spatial links between nearby patches. On a four-class and a two-class classification, the model obtained classification accuracy of 0.95 and 0.99, respectively. The cancer genome atlas (TCGA) and the cancer imaging archive (TCIA), each containing 2034 and 2005 basic pictures, respectively, were used to train the algorithm.

To categorize glioblastoma (GBM) and low-grade glioma (LGG) pictures, Yonekura et al.^24^ presented a deep CNN (DCNN) model with 14 layers. 200 H &E histopathological WSIs from TCGA make up the dataset. Ten thousand unique patches are retrieved from each cohort and used as training data for their model. To evaluate performance, common DCNN architectures, including LeNet^25^, ZFNet^26^, and VGGNet^27^ were tested, and the results were contrasted with those of the suggested model. Based on WSIs, Kolachalama et al.^28^ predicted the survival rates of kidney tumors by using DCNN. Three classes of survival rates $1 year, 3 years, and 5 years$ were produced using their model, with outcomes of 87%, 87%, and 90%, respectively. They did not extract any patches, which was a computationally intensive process. They used WSIs as inputs.

Deep survival convolutional network (DeepSurvNet) was introduced by Zadeh Shirazi et al.^29^ to reliably classify the survival rates of brain cancer patients. Based on H &E histopathology images, the model gives survival likelihood ranges across four classes. The datasets were obtained from the TCGA and a nearby hospital. They included 450 H &E slides from individuals with various types of brain tumors. For patients who had survived for 0–6, 6–12, 12–24, and over a year, they took into consideration classifications of four grades. The TCGA dataset was used to train and evaluate DeepSurvNet, which is built on the GoogLeNet^30^ architecture. A private dataset was also used to generalize DeepSurvNet. In the testing phases, their model obtained precisions of 99% and 80%. They also examined the frequency of genes linked to each class.

Liu et al.^31^ employed DCNN to predict the mutational status of isocitrate dehydrogenase (IDH), an essential biomarker in glioblastoma. 266 H &E slides with grade 2–4 gliomas were gathered for their dataset from the TCGA and a private hospital. They presented a model based on using Resnet50 DCNN architecture as the primary structural support for IDH status prediction and generative adversarial networks (GAN) to produce synthetic samples to enable data augmentation. They also inferred that the DCNN model could predict IDH status more precisely when patients’ ages are added as a new feature. They could identify the IDH mutational status with an accuracy of 85%.

With accuracy rates of 83.25% and 82.1%, respectively, Bayramoglu et al.^32^ developed two CNN architectures for the detection of malignancy in breast cancer. On each magnification factor of the BreaKHis dataset, they evaluted their performance. Using the BreakHis dataset, Sudharshan et al.^33^ created a supervised learning system that, on average, had 82.67% accuracy. They also looked at the applicability of multiple instance learning for the categorization of breast cancer. Using a pre-trained CNN network on the BreakHis database, Alrahhal^34^ was able to detect breast cancer in histological images with an accuracy of 86.4%, whereas Truong and Pham^35^ evaluated their CNN architecture and achieved 77.3% accuracy.

To prevent overfitting, DL models like CNNs need to be trained optimally on a lot of balanced, labelled data. DL techniques are frequently used in the analysis of histopathological images^36^. While Hou et al.^37^ proposed a patch-based CNN with an expectation-maximization technique. The sparse autoencoders have been used in^38^ to extract features from histopathology files. Zheng et al.^39^ presented a CNN-based nuclei-guided feature extraction method for histopathology imaging.

Amin et al.^40^ suggested two phases approach. They first segmented breast lesions using a model made up of deepLabv3 and Xception models that had already been trained. A few chosen parameters were used to train the model, greatly enhancing the segmentation of breast cancer. Second, a classification model with six layers and four qubits was used. Salman et al.^41^ created a system that recognizes carcinogenic areas in tissue pictures and assigns them a grade using the Gleason grading system. They used 450 actual biopsy pictures to retrain a Yolo identification model built on CNN with over 1800 annotated prostate tissue. For 24 hours, the system was tuned with default hyperparameters up until the loss function dropped below 5%. Three pathologists examined each discovered region’s accuracy after testing it on two sets of biopsy pictures.

Chan et al.^42^ applied two neural networks, VGG16 and Resnet50, to process the WSI with feature extraction. They used k-means and random forest methods to categorize the three forms of brain malignancies (glioblastoma, oligodendroglioma, and astrocytoma). They compared prediction results with and without magnetic resonance imaging (MRI) characteristics during the prediction stage. Pei et al.^43^ proposed a DL-based technique for brain tumor classification, which was divided into two parts. The first step was to segment a brain tumor on a multimodal MRI. The second step was classifying the tumors based on the tumor segmentation results. A 3D deep neural network (DNN) is implemented to distinguish tumors from normal tissues, followed by the development of a second 3D DNN for tumor categorization.

Pei et al.^44^ developed a DNN-based method for brain tumor classification and grading based on histology and genetic data, utilizing the most recent world health organization (WHO) classification criteria from 2016. To increase performance, the classification approach incorporated a cellularity characteristic obtained from the morphology of brain tumor histology images. By employing the over-segmentation technique, they also presented a region of interest (ROI) selection strategy for histopathological WSIs. Lakshmi et al.^45^ used a DL method called the Inception-v3 model. The softmax classifier was utilized in their approach to categorize the photos into various classes. The Adam optimizer and loss function were used to optimize the network settings.

Attallah ^46^ proposed a technique called CoMB-Deep classifying medulloblastoma from histopathology pictures. Ten CNNs are used for complex feature extraction, discrete wavelet transform is integrated for fusion and dimension reduction, and Bi-LSTM networks are used for improved classification.Even though CoMB-Deep shows encouraging results, there are still important factors to take into account. The pipeline’s complexity could be increased by integrating ten CNNs and then fusing features with discrete wavelet transform (DWT). Longer training times, scalability issues, and computational inefficiency could result from this. Attallah and Zaghlool ^47^ introduced textural images derived from GLCM and GLRM texture analysis methods. Using both original and textural images, three deep learning models (ResNet-101, Inception, and InceptionResNet) were trained to extract deep features.

Mohan and M ^48^ extracted localized pathology features by analyzing individual smaller tile pictures. From these tiles, they extracted features such as the Gray Level Co-occurrence Matrix, Histogram(GLCM), Gabor, and Perceptual Features(PF). For the purpose of assessing performance, these multidimensional feature sets are subsequently fed into classifiers like KNN, SVM, Naive Bayes, and Logistic Regression. The methodology’s reliance on a combination of diverse feature extraction methods (PF, Histogram, Gabor, GLCM) might introduce redundancy or irrelevant features, potentially impacting model performance. The challenge of selecting the most informative and relevant features among these diverse sets could impact the model’s robustness and generalization.

Im et al. ^49^ trained the ResNet50V2 model to classify diffuse glioma grades and subtypes in a deep transfer learning framework using clinical-grade pathological images. The model demonstrated promising results in subtype classification, with a commendable accuracy of approximately 87.2% in differentiating between glioma subtypes. Satyanarayana et al. ^50^ presented a CNN with mass correlation analysis for feature extraction and weight assignment. The lack of information in the paper regarding the validation process and the potential biases introduced by the preprocessing steps.

Archana and Komarasamy ^51^ presented a method that uses a Bagging Ensemble with K-Nearest Neighbor (BKNN) for classification and U-Net for image segmentation. Ozer et al. ^52^ applied a resnet50 deep neural network to analyze cytological images taken during surgery. Twenty-five medical images from squash smear slides were used in the study. These images showed samples of non-neoplastic brain tissue as well as high-grade and low-grade gliomas, as well as metastatic carcinomas. 5-fold cross-validation was used for the neural network’s training and assessment. The model performed with a 95% diagnostic accuracy at the patch-level classification. Despotovic et al. ^53^ introduced a detailed comparison of deep learning architectures and transfer learning techniques for the classification of adult-type diffuse gliomas. The generalizability of ImageNet representations to histopathological images is assessed, pretraining methods such as self-supervised and multi-task learning are investigated, and a semi-supervised strategy utilizing weak labels is presented to enhance model performance. Table 1 provides an overview of some recent related work.

**Table 1:** A summary of some current related work.

Study	Analysis type	Methodology	Performance
Sumi et al.^23^	Classification for benign, Oligodendroglioma, and GBM	The InceptionResNetV2 is used to extract hierarchical features. A deep spatial fusion network was built to extract spatial features from across patches	ACC = 95%
Yonekura et al.^24^	Classification for GBM	Built a DCNN model with 14 layers. Feature descriptors and a classification strategy are simultaneously acquired using DCNN	ACC = 96.5%
Shirazi et al.^29^	Classification for brain cancer patients’ survival rate	DeepSurvNet is the suggested classifier. DeepSurvNet is a GoogleNet classifier that was developed using the TCGA dataset	Precision = 99%
Liu et al.^31^	Determination of the IDH mutational status of gliomas	A data augmentation technique based on the GAN methodology were presented for the prediction of IDH mutational status using H &E slides	ACC = 85.3%
Bayramoglu et al.^32^	Benign/Malignant Classification	Single-task CNN and multi-task architectures are presented. CNN is employed to concurrently classify the malignancy and the degree of image magnification. The suggested methodology allowed the integration of picture data from many resolution levels	ACC = 83.25%
Chan et al.^42^	Discrimination between glioblastoma, oligodendroglioma, and astrocytoma	Two neural networks, VGG16 and Resnet50, was utilized to process the WSIs and extract features. K-means and random forest (RF) approaches are applied to categorize brain malignancies	DSC = 89%
Pei et al.^44^	Automated Glioma Grading	Shape-based measurement for aberrant cell nuclei was applied. The performance of the DNN-based classification approach is then enhanced by combining cellularity and molecular characteristics	ACC = 93.81%
Lakshmi et al.^45^	Brain Tumor Detection	The Inception-v3 model was used. Their model extracts the multi-level features and categorizes them	ACC = 89%

According to the above description, some of the present related work’s limitations are highlighted, which can be summarized as follows: First, some research only included two or three grades of brain tumors (glioblastoma, oligodendroglioma, and astrocytoma), which may not be an accurate representation of brain tumors in general. Second, many studies relied on subjective and time-consuming manual tumor segmentation. Third,There were significant challenges for some methods including potentially missing global context, difficulties in capturing subtle tumor features. Fourth, some techniques require two distinct 3D DNNs for tumor identification and classification, which can be computationally intensive and may require a large amount of data. Finally, some approaches only used one deep learning model, which might not be adequate for correctly identifying more complex cases.Our suggested method aims to address these drawbacks. Our goal is to increase the model’s generalizability and enhance subtle feature recognition by fusing the accuracy of YOLOv5 with the feature extraction capabilities of ResNet50.

To address the limitations of the existing literature and enhance the diagnosis performance of identifying different grades of brain tumors, we developed a CAD system based on the hybrid YOLO and RestNet50 DL model.The advantage of integration between yolov5 and resnet50 is its robustness to variations in image quality, tumor size, and location which addresses the limitation of some related work. We began by preprocessing the photos. The primary purpose is to improve contrast and reduce noise from entered photos. Then, we used various processing techniques to make all photos the same size and increase their number. All photos from the used datasets were shrunk to (256,256), cropped, rotated, and color normalized. The standard RestNet50 was then tweaked its hyperparameters to appropriately diagnose the various grades. We compared the suggested model to some cutting-edge models. We used five different performance criteria in the comparison to evaluate the model’s performance in diagnosing healthy and varied Glioma grades.

## The proposed framework

**Figure 2:**
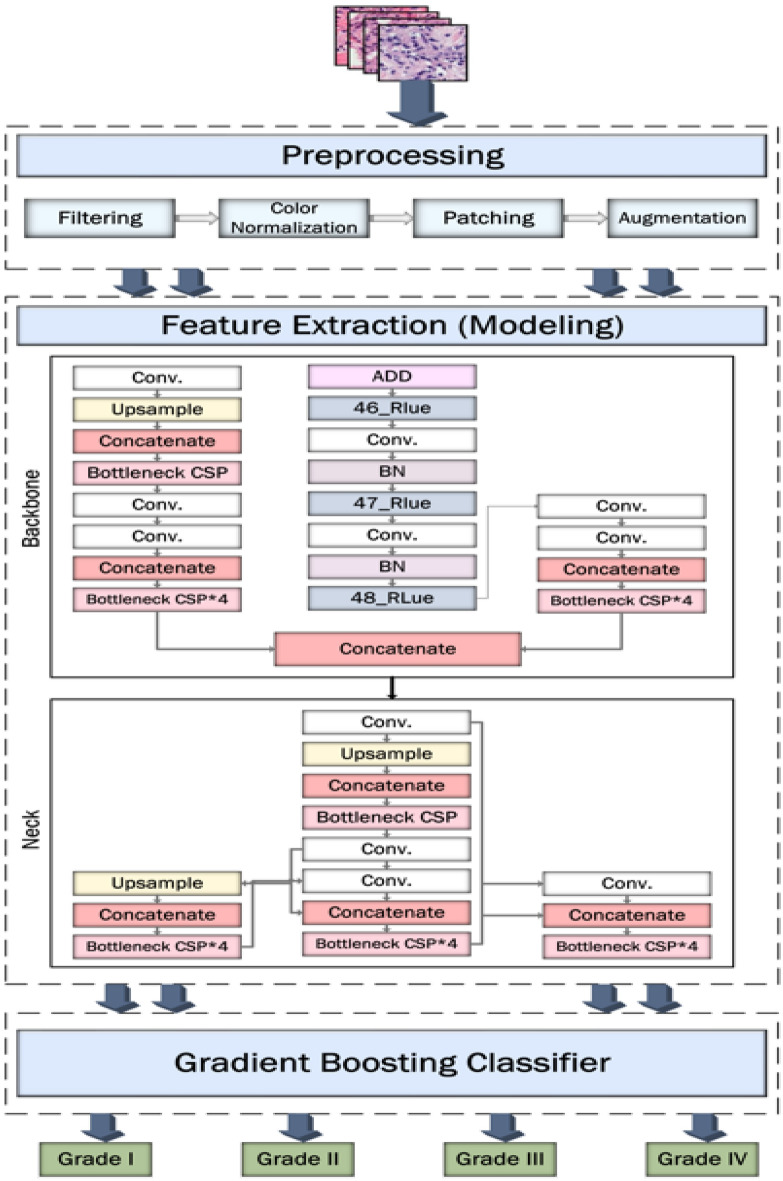
The proposed framework.

Transfer learning is widely used in a variety of contexts. Pre-trained models detect simple features like shapes and diagonals in the first layer. In the subsequent layers, they combine these elements to pick up multipart features. In the final layer, the models create meaningful constructs by exploiting features discovered in previous stages. We use two well-known models to extract features, which are then used to train the models. Figure 2 depicts an overview of the study’s workflow.

In order to address the complex challenges of brain tumor grading in large-scale histopathological images, this work presents a new hybrid model that combines the strengths of three powerful architectures. By deliberately combining:

- *YOLOv5:* well known for its accurate and efficient object detection capabilities, which allow for the precise localization of tumors within large histopathological WSIs.
- *ResNet50:* a deep CNN with strong feature extraction capabilities that offers detailed representations of complex tumor properties required for accurate grading.
- *XGBoost:* an effective gradient boosting classifier that can further improve classification accuracy by capturing complex non-linear interactions and high-dimensional features within the extracted features.

This innovative combination offers a number of benefits and constitutes a noteworthy contribution to the field:

- *Enhanced Feature Representation:* By combining the strengths of YOLOv5 and ResNet50, a more comprehensive and insightful feature representation of the tumor is produced, including contextual and spatial information that is essential for a precise diagnosis.
- *Improved Grading Accuracy:* The object detection capabilities of YOLOv5 enable accurate tumor localization within the WSI, whereas the deep feature extraction of ResNet50 enables the identification of subtle tumor characteristics, resulting in improved accuracy for grading task.

### Preprocessing

Because whole slide photos cannot be handled directly due to their vast size, some preprocessing processes must be taken. It is also necessary to account for the variety in stain colors across the dataset while ignoring the glass background of the scans. The Open Slide program was used to access all the image slides^54^.

#### Filtering

WSIs often have at least a 50% white background, as the white background in WSI analysis carries no useful information and can obstruct classification. Background filtering is a crucial preprocessing step. Different techniques have been tried to get rid of the background, such as color thresholding and morphological operations. This model uses a straightforward color thresholding technique that examines each pixel’s green channel values and eliminates any that are more than the 200-intensity threshold. When a binary mask is produced, if it covers more than 90% of the image, the threshold is adjusted until it only covers less than 90%. This challenge was completed using the adaptive non-local means thresholding algorithm^55^. When using H & E-stained histopathological images, where tissue has less green content in comparison to the white backdrop, this approach performs well.

It is crucial to note that the filtering procedure might also eliminate pixels inside the tissue area that have a brighter color. The median filter, closing filter, and holes filling algorithm were used to eliminate small holes left by the background filter in order to solve this problem. The filtering procedure was also used on the WSI thumbnails, which are the smallest-resolution representations of the full-sized images, to save computing time. The generated binary mask was then upscaled to its full resolution. While maintaining the accuracy of the segmentation and classification results, this method can significantly lower the computational cost of the background filtering procedure.

#### Stain normalization

WSIs in the TCGA dataset were prepared at multiple clinics and stained using a variety of H &E compounds, which can cause variances in stain colors due to environmental factors. Stain normalization is regarded as an important preprocessing step because the variability in stain colors can make it challenging for a neural network to discriminate between GBM and LGG classes^56^. In this study, stain normalization uses the Vahadane algorithm^57^, which effectively maintains biological structures.

In four cancer datasets, Roy et al.^58^ determined that Vahadane’s method was the best color normalization technique for histopathology images. For normalizing source images without color distortion, the algorithm needs a target image. From the original photographs, stain density maps are generated, capturing the relative concentrations of the two stain colors, which provide crucial details about the biological structures. The target image’s stain color foundation is mixed with the density maps to change only the colors while maintaining the intensity of the structures. The target image was chosen rather arbitrarily to encompass a wide range of colors from the dataset. Each image is normalized independently since removing the white pixels of the backdrop first is recommended because they are simply formed of the two base stain colors.

#### Patching

One popular method for getting around the restrictions of applying neural networks to analyze huge images is to use small patches. Choosing the right patch size is essential to balance the analysis’s resolution and processing needs. A patch size of 1024 x 1024 at 20x magnification makes sense in the context of WSI analysis for tumor detection since it corresponds to the size skilled pathologists can use to identify tumors. When this size is scaled down to the input size anticipated by the majority of CNNs trained on the ImageNet dataset, which is typically 224 $\times$ 224 pixels, the area is significantly reduced^59^. Based on these results, the current study attempted two patch sizes, small (256 $\times$ 256) and large (512 $\times$ 512), and successfully extracted a total of 225,213 small patches and 40,114 large patches that did not overlap using the tiling technique^60^. To prevent analytical redundancy and guarantee the independence of the patches, it is crucial to use non-overlapping patches. We utilized a variety of augmentation techniques to enhance the diversity and resilience of the dataset by adding to the available data samples. A variety of transformations, such as rotation, scaling, flipping, translation, and adjustments to brightness and contrast, were included in these augmentation techniques. Furthermore, we used geometric transformations to simulate different viewing angles and perspectives, such as affine transformations.Table 2 summarizes the number of extracted patches of various sizes for each class.

**Table 2:** Number of patches for each class.

	No. of ROIs	No. of patches (256$\times$256)	No. of patches (512$\times$512)
Class I	221	50,621	8939
Class II	215	54,440	9660
Class III	282	75,942	13,575
Class IV	152	44,210	7940
Total	870	225,213	40,114

### Modeling

This section discusses our Glioma classification algorithms. We apply the two-step technique. The initial stage is target detection using standard approaches, such as YOLOv5. The image classifier is used to perform classifications in the second stage. Similar to^61^, the decision to utilize a ResNet50 model was chosen because it has already demonstrated its capability in medical image processing. The prediction performance of a pre-trained network is compared to that of a CNN constructed from scratch in this study.

#### ResNet50

The residual network is referred to as ResNet. It forms a crucial component of the traditional computer vision task, which is crucial for target classification. ResNet50, ResNet101, and so on are examples of the classic ResNet. The issue of the network developing in a deeper direction without gradient explosion is resolved by forming the ResNet network. As is well known, DCNNs are excellent at extracting low-, medium-, and high-level characteristics from images. We can typically improve accuracy by adding additional layers. The activation function of each of the two dense layers in the residual module is the ReLU function. Because ResNet-50 offers the benefits of lower input complexity, computational efficiency, and pre-trained weight availability, we deliberately chose it over ResNet-152, even though the latter may have improved accuracy. By utilizing the optimal depth of ResNet-50 for whole-slide image analysis, it was possible to process a greater number of patches for reliable analysis and effectively concentrate on specific features of glioma tumors while preserving essential information. ResNet-50 was chosen as a practical and effective option for brain tumor grading because it was highly efficient in our training process and gave a strong foundation for feature extraction when we used pre-trained ResNet-50 weights.

Deep CNN ResNet-50 has a lightweight design. There are 50 layers that reformulate learning residual functions concerning the layer inputs rather than learning unreferenced functions. The ResNet concept comprises a stack of related or “residual” pieces. This block represents an array of convolutional layers. An identity mapping path also links a block’s output to its input. The channel depth is increased while stride convolution continually downscales the feature mapping to maintain the time complexity per layer.

#### YOLO

YOLOv5 is a compelling option for a dependable and effective detection model because of its greater maturity, ease of use, availability of pre-trained models, optimized performance for real-time applications, and low resource requirements, even though there are newer YOLO versions with possibly higher accuracy. Because YOLOv5’s architecture is less complex and requires less resources than its more recent versions, it can analyze large datasets effectively on standard computing hardware. This is critical for the analysis of large amounts of histopathological data without the need for costly or specialized equipment. In order to analyze large datasets of histopathological images in real-time and greatly increase workflow efficiency, YOLOv5’s speed is very important. Newer versions may be marginally more accurate, but in this case, their slower inference speed makes them less useful. Although there is a greater theoretical accuracy with YOLOv6-v8, YOLOv5 is a more practical and advanced option for brain tumor detection in histopathological images due to its established presence in medical research, pre-trained medical models, ease of use, resource efficiency, and integration capabilities. With its adaptability to handle various tumor types with little effort, and its speed and real-time inference capabilities, it is an invaluable tool for analyzing large datasets.

**Figure 3:**
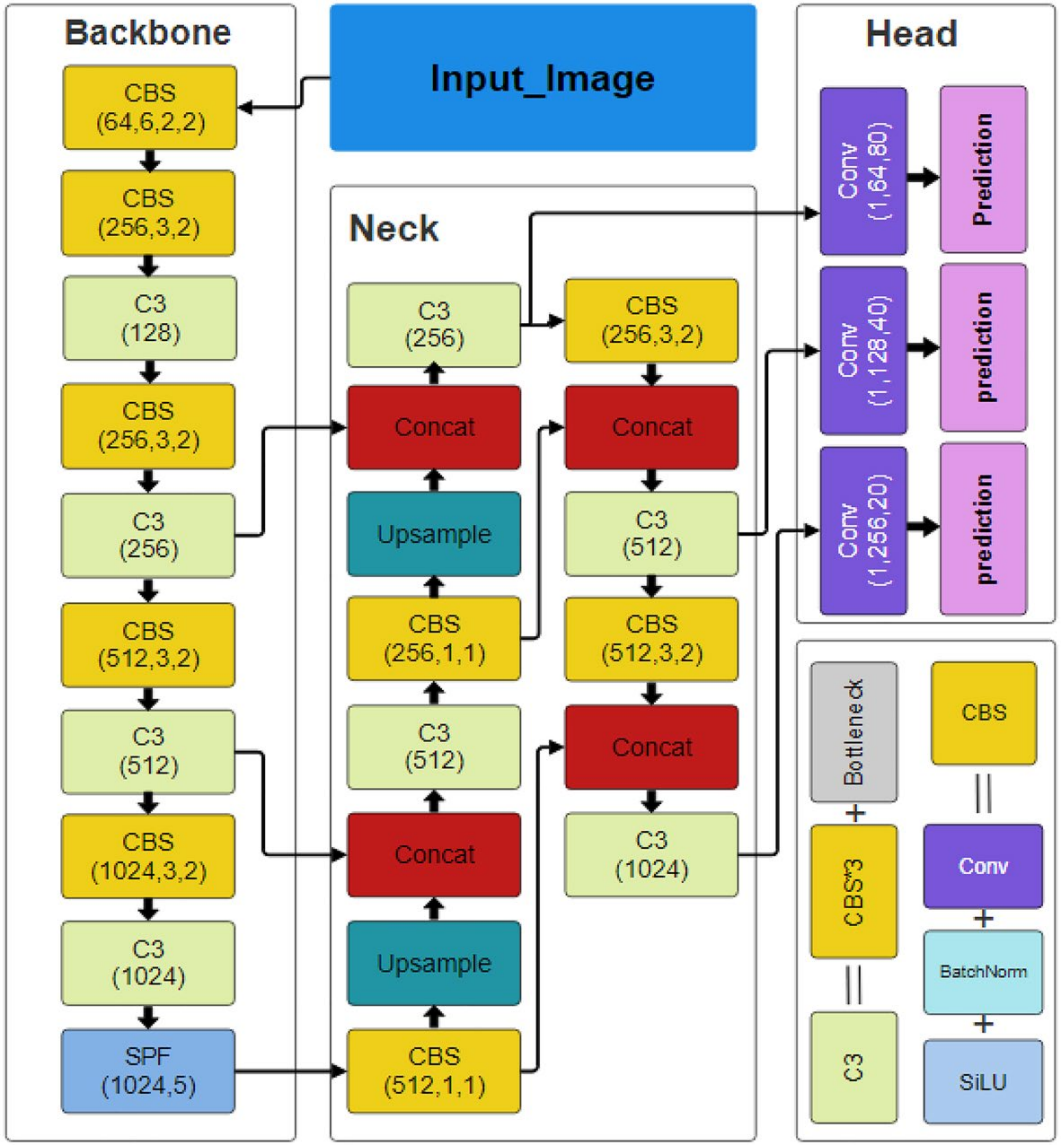
The architecture of YOLOv5 model.

The backbone, neck, and head comprise the three essential structural components of the YOLO series of models. The original architercture of Yolov5 is shown in Fig. 3. CSPDarknet is used by YOLOv5 as the backbone to extract features from photos composed of cross-stage partial networks. In the YOLOv5 neck, the features are aggregated using a feature pyramid network created by PANet, which is then sent to the head for prediction. Concerning object detection, the YOLOv5 head has layers that produce predictions from anchor boxes. In addition, YOLOv5 chooses the following options for training^62^:

- Leaky ReLU and sigmoid activation are used by YOLOv5, while SGD and ADAM are available as optimizer alternatives.
- Binary cross-entropy is used for logit loss as the loss function

YOLOv5 has a variety of pre-trained models. The trade-off between model size and inference time is what separates them. Although only 14MB in size, the YOLOv5s lightweight model is not very realistic. On the other end of the range, we have the 168MB-sized YOLOv5x, which is the most accurate member of its family. YOLOv5 features several lighting spots over the YOLO series, including:

1. Multiscale: To improve the feature extraction network, employ the FPN rather than the PAN, resulting in a simpler and quicker model.
2. Target overlap: The target can be mapped to several nearby central grid points using the rounding method.

**Figure 4:**
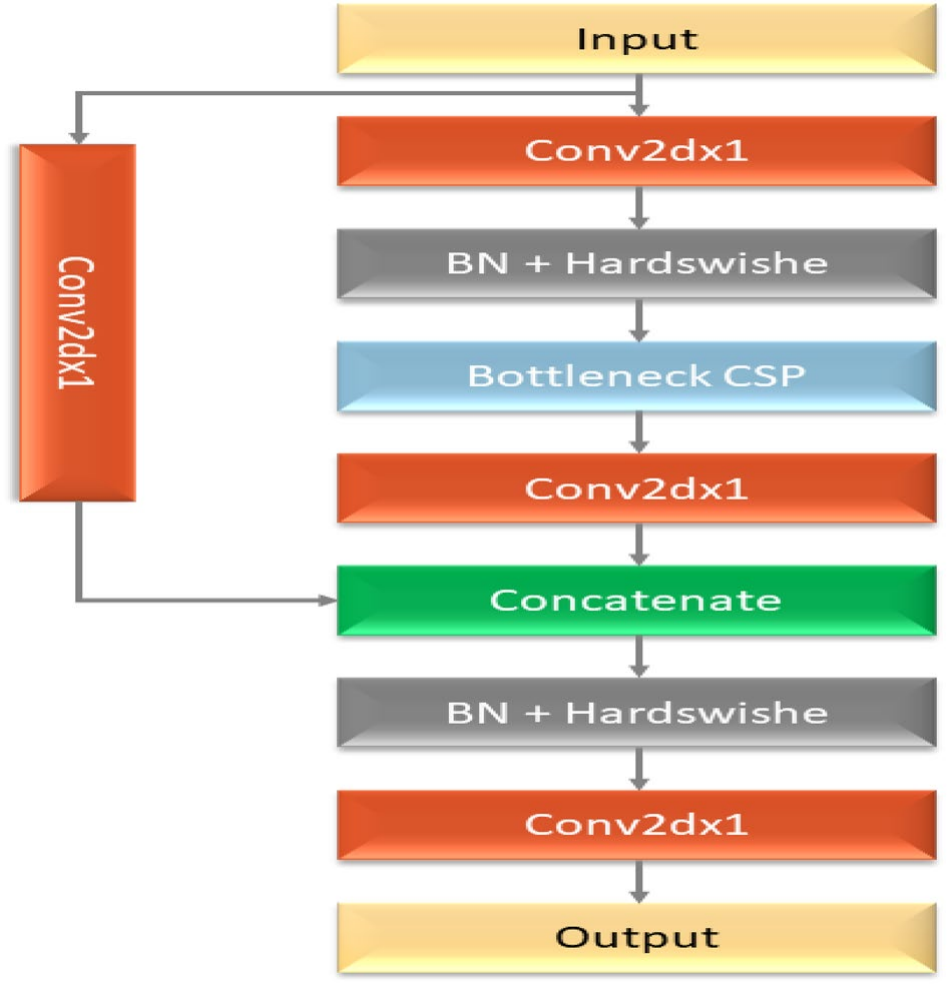
The architecture of bottleneck model.

The fundamental purpose of the model backbone is to extract essential characteristics from an input picture. The backbone network’s first layer, called the focusing layer, is utilized to speed up training and simplify model calculations. The following objectives are achieved by it: The three-channel picture is divided into four slices for each channel using a slicing method. The output feature map was generated using the convolutional layer comprised of 32 convolution kernels. Then, the four sections are connected in depth using concatenation, with the output feature map having a size of. After that, the results are output into the next layer using the Hardswish activation functions and the batch normalization (BN) layer. The third layer of the backbone, the BottleneckCSP module, was created to efficiently extract in-depth information from the picture. The Layer (Conv2d + BN + ReLu) with a convolution kernel size is joined to produce the Bottleneck module, which is the main component of the BottleneckCSP module illustrated in Fig. 4. The ultimate output of the bottleneck module is the result of adding the output of this portion to the original input obtained through the residual structure.

The operation of the CSP network from the first layer to the last layer is shown by Eqs. 1–3.

1 $$\begin{aligned} & L_{1}= W_{1} x L_{0} \end{aligned}$$

2 $$\begin{aligned} & L_{2}= W_{2} x [L_{0},L_{1}] \end{aligned}$$

3 $$\begin{aligned} & L_{k}= W_{k} x [L_{0},L_{1},L_{2},\ldots ,L_{(k-1)} ] \end{aligned}$$

where $[L_0, L_1,\ldots ]$ means concatenating the layer output, and $W_i$ and $L_i$ are the weights and output of the i-th dense layer, respectively. Three components make up the YOLO loss function: classification error, intersection over union (IOU) error, and coordinate prediction error. The coordinate prediction error shows the precision of the bounding box’s position, which is defined by Eq. 4.

4 $$\begin{aligned}&Error_{Cod} = \lambda _{Cod} \sum \nolimits _{i=0}^{G^2} \sum \nolimits _{j=1}^{N} I_{ij}^{T} [(a_{i}- \bar{a_{i}})^{2} + (b_{i}- \bar{b_{i}})^{2}] \\&\quad +\lambda _{Cod} \sum \nolimits _{i=0}^{G^2} \sum \nolimits _{j=1}^{N} I_{ij}^{T}[(w_{i}- \bar{w_{i}})^{2} + (h_{i}- \bar{h_{i}})^{2}] \end{aligned}$$

where $\lambda _{Cod}$ represents the weight of the coordinate mistake in Eq. 4. $G^{2}$ represents each detection layer’s total number of grid cells. N represents the total number of bounding boxes in each grid cell. If a target is present within the j-th bounding box of the j-th grid cell, $I_{ij}^{T}$ will signal this. $\bar{a_{i}}, \bar{b_{i}}, \bar{w_{i}}, and \bar{h_{i}}$ denote the anticipated box, whereas $a_{i}, b_{i}, and w_{i}, h_{i}$ denote the abscissa, ordinate, width, and height of the center of the ground truth, respectively.

The intersection over union (IOU) error shows how closely the predicted box and the ground truth intersect. Eq. 5 provides a definition.

5 $$\begin{aligned}&Errror_{IOU}= \sum \nolimits _{i=0}^{G^2} \sum \nolimits _{j=1}^{N} I_{ij}^{T} (C_{i}- \bar{C_{i}})^{2} \\&\quad + \lambda _{notar} \sum \nolimits _{i=0}^{G^2} \sum \nolimits _{j=1}^{N} I_{ij}^{notar} (C_{i}- \bar{C_{i}})^{2} \end{aligned}$$

The confidence cost in the absence of an object is described by the notation “$\lambda _{notar}$” in Eq. 5. The confidence in the truth and prediction are denoted by $C_i$ and $\bar{C_i}$. In Eq. 6, the term “classification error” is used to describe the accuracy of categorization.

6 $$\begin{aligned} Errror_{CL}= \sum \nolimits _{i=0}^{G^2} \sum \nolimits _{j=1}^{N} I_{ij}^{T} \sum \nolimits _{c\in class} (p_{i}(c)- \bar{p_{i}}(c))^{2} \end{aligned}$$

The discovered target’s class is denoted by the letter *c* in Eq. 6. The genuine probability that the target is in class c is denoted by the $p_{i}(c)$ symbol. The estimated likelihood that the target is a member of class *C* is denoted by the $\bar{p_{i}}(c)$ symbol. So, Eq. 7 represents the definition of the YOLO loss function.

7 $$\begin{aligned} \begin{aligned} Loss&= \lambda _{Cod} \sum \nolimits _{i=0}^{G^2} \sum \nolimits _{j=1}^{N} I_{ij}^{T} [(a_{i}- \bar{a_{i}})^{2} + (b_{i}- \bar{b_{i}})^{2}] \\&\quad +\lambda _{Cod} \sum \nolimits _{i=0}^{G^2} \sum \nolimits _{j=1}^{N} I_{ij}^{T}[(w_{i}- \bar{w_{i}})^{2} + (h_{i}- \bar{h_{i}})^{2}] \\&\quad +\sum \nolimits _{i=0}^{G^2} \sum \nolimits _{j=1}^{N} I_{ij}^{T} (C_{i}- \bar{C_{i}})^{2} \\&\quad + \lambda _{notar} \sum \nolimits _{i=0}^{G^2} \sum \nolimits _{j=1}^{N} I_{ij}^{notar} (C_{i}- \bar{C_{i}})^{2} \\&\quad +\sum \nolimits _{i=0}^{G^2} \sum \nolimits _{j=1}^{N} I_{ij}^{T} \sum \nolimits _{c\in class} (p_{i}(c)- \bar{p_{i}}(c))^{2} \end{aligned} \end{aligned}$$

#### Improved YOLOv5

**Figure 5:**
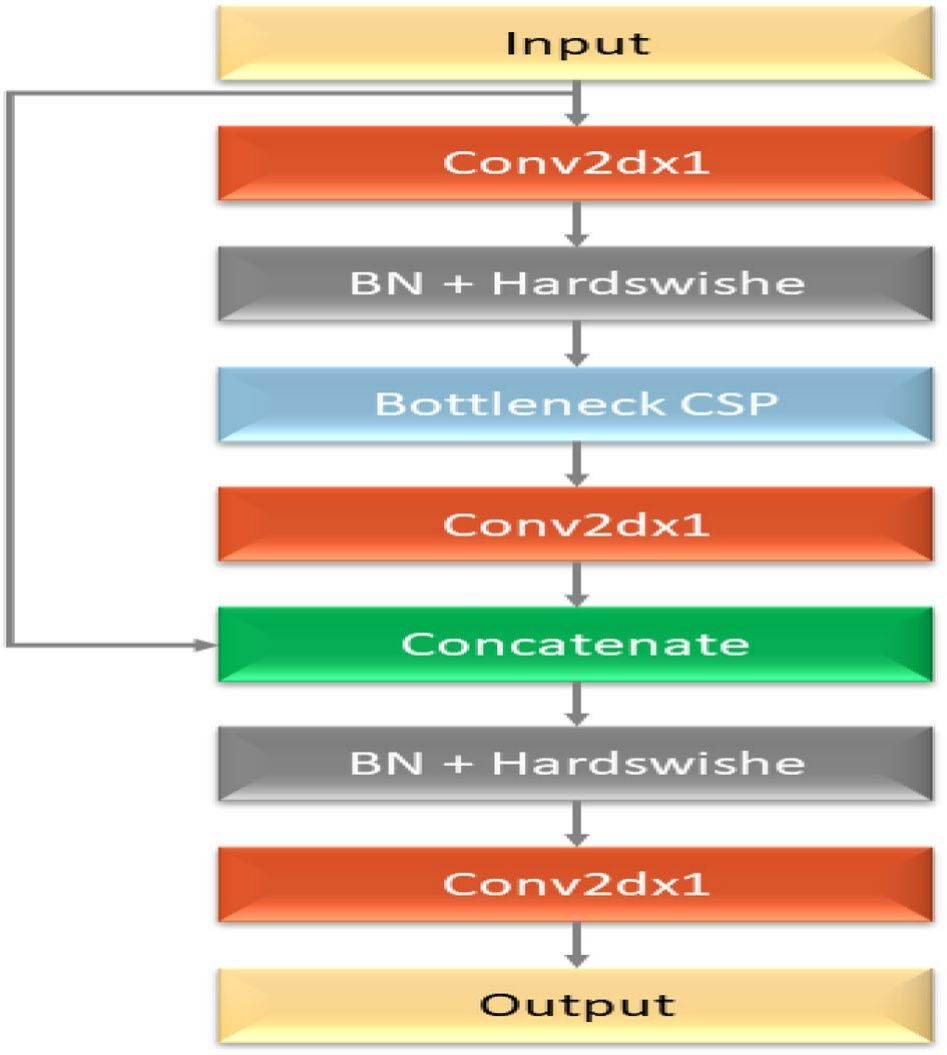
The modified bottleneck.

The YOLOv5 model’s initial implementation does not lead to the intended outcomes. The model should accurately identify and classify cancers, even on intricate surfaces. In order to implement the model in hardware devices, its size must also be as small as feasible. We thus modify the model’s skeleton in a few ways. The YOLOv5 architecture’s core network comprises four BottleneckCSP modules, each having several convolutional layers. Even though the convolution process may extract picture information, the convolution kernel has many parameters, which also leads to many parameters in the recognition model. The consequence is deleting the convolutional layer on the alternative branch of the original CSP module. The input and output feature maps of the BottleneckCSP module are linked directly by another branch in-depth, significantly decreasing the number of parameters in the module. Figure 5 depicts the architecture of the enhanced BottleneckCSP module. The Optuna library has used to implement hyperparameter tuning for this layer. A summary of our methodology is provided below:

- Defined Search Space: We created a search space with the following parameters: activation function (ReLU, Leaky ReLU, Swish), number of filters (128–192), and kernel size (1–3).
- The primary objective function used to assess each configuration was validation accuracy.
- Trial Budget: To balance exploration and exploitation within the Optuna framework, we set a trial budget of 50 training runs.

The following are the outcomes of the hyperparameter tuning: The best-performing configuration was determined by Optuna to include 154 filters, a kernel size of 3, and Leaky ReLU activation.

#### The whole structure of the model

The RESNET-50 AND YOLOV5 fusion technique uses the result of one of ResNet-50’s layers as an input to the YOLO neck while combining it with the result of enhanced bottleneckCSP. This ResNet-50 network layer is designated as a feature extraction layer in YOLO. In this study, the feature extraction layer was based on the ReLU (activation 49 ReLU) layer. The remaining layers of ResNet-50, which include the average pooling, fully connected, softmax, and classification layers, are shortened and combined with the YOLO layer to create a new fused network architecture for the detection and classification of brain tumors, as presented in Fig. 1. We processed the source photos before feeding them into YOLOv5 and ResNet50. YOLOv5’s backbone was enhanced by the integration of ResNet50 as a complementary feature extractor to achieve the best feature extraction for the classification within the architecture. We recognized that the ResNet50 model could capture high-level visual representations, so we initially trained it with weights pretrained on ImageNet by utilizing transfer learning principles. We tuned the last few layers of the ResNet50 model specifically to meet the requirements of our detection task in the YOLOv5 framework. In order to fine-tune the network, most of its layers were frozen, and the weights of the fully connected and final convolutional layers were changed. Our goal in fine-tuning these chosen layers was to modify ResNet50’s feature extraction capabilities so that they more closely matched the traits and intricacies present in our object detection dataset. This fine-tuning approach not only enhanced the model’s capacity to extract complex visual features pertinent to our task, but it also expedited convergence when the YOLOv5 detector was subsequently trained. By concatenating the outputs from the YOLOv5 backbone with the activations from the final two layers of ResNet50, we performed a targeted feature fusion to maximize the strengths of both models. With a focus on semantic richness, the deeper layers of ResNet50 extracted detailed feature maps that were strategically fused into the spatial hierarchy captured by YOLOv5. After this combination, we took a fine-tuning strategy, focusing on the joined layers to enable a well-balanced combination of feature representations from both networks. We specifically started selectively fine-tuning the concatenated layers so that the original YOLOv5 architecture wouldn’t be disturbed, and we could gradually adapt to the specifics of the detection task. To facilitate a more customized extraction of discriminative features relevant to the intricacies of our dataset, this fine-tuning mainly involved modifying the weights and biases of the combined layers.Through the process of feature concatenation and fine-tuning, we were able to combine the strengths of both ResNet50 and YOLOv5 in order to maximize their complementary abilities. This method not only accelerated the model’s ability to capture context and fine-grained visual details, but it also made improved the classification performance possible. XGBoost categorizes the input data and creates predictions when features from a hybrid model have been extracted. Since XGBoost is better at handling high-dimensional features, capturing non-linear relationships, and adapting to different tumor presentations, we chose them over classical machine learning models. This combination of parameters contributes to the success of improving algorithms in medical image analysis and ensures effective analysis and diagnosis in clinical settings by enabling robust and accurate classification of brain tumors in histopathological images. By applying XGBoost as a classifier, we may benefit from its capacity to manage big datasets and intricate feature spaces, making it an excellent option for this kind of task. Overall, the predictions produced by combining XGBoost with ResNet50 and YOLOv5 are more precise and effective. Algorithm 1 lists the glioma tumor classification technique based on brain histopathological RGB images.

**Algorithm 1:**
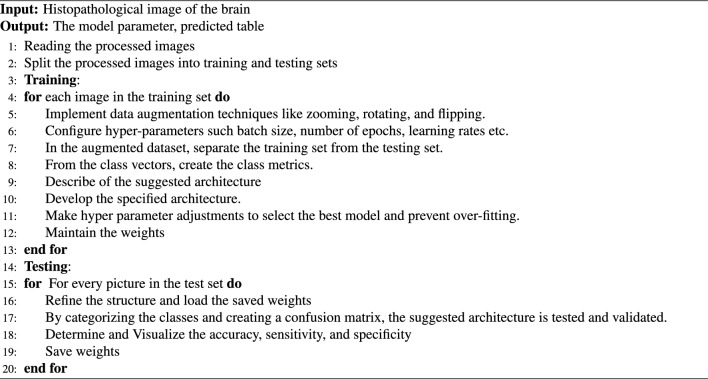
The glioma tumor classification

## Experimental results

### Dataset description

937 WSIs from 490 patients with brain cancer were retrieved from TCGA. The dataset is described in Table 3. The WSIs were visually examined, and those that were determined to be useless because of corruption, irremovable marker markings, poor resolution, or a lack of clinical information were eliminated. This led to 445 cases with 654 WSIs that could be examined further.

**Table 3:** The characteristics of TCGA dataset.

Characteristics	Complete dataset	LGG	HGG
No. of patients	490	184	306
No. of deaths	316	94	222
Age	49.65	40.37	56.79
Gender			
Male	303	115	188
Female	187	69	118

### Performance metrics

In this section, we will review some of the evaluation metrics we employed in our experiment. We know that detection is the most critical aspect of the CAD system. Accuracy (ACC), sensitivity, specificity, receiver operating characteristic (ROC) curve, overlapping error, boundary-based evaluation, and the Dice similarity coefficient (DSC) are common metrics for analyzing the effectiveness of classification systems. The following equations can be used to calculate ACC, sensitivity (SEN), specificity (SPC), positive predictive value (PPV), and DSC.

8 $$\begin{aligned} & ACC = \frac{TP + TN}{TP + TN + FP + FN} \end{aligned}$$

9 $$\begin{aligned} & SEN = \frac{TP}{TP + FN} \end{aligned}$$

10 $$\begin{aligned} & PPV = \frac{TP}{TP + FP } \end{aligned}$$

11 $$\begin{aligned} & SPC = \frac{TN}{TN + FP } \end{aligned}$$

12 $$\begin{aligned} & DSC = \frac{2 TP}{2 TP + FP + FN} \end{aligned}$$

It is possible to calculate the Pearson product-moment correlation coefficient between expected and observed values using the Matthew correlation coefficient (MCC), which is unaffected by the problem of unbalanced datasets. The percentage of correctly identified tumor and non-tumor samples in the categorization of brain tumors using histopathological images is the ACC. SEN would be the model’s capacity to accurately classify tumor samples as positive considering their histological characteristics. The model’s SPC would be its capacity to appropriately classify non-tumor samples as negative based on their histological characteristics. PPV is the percentage of tumor samples with a histological diagnosis that the model correctly identifies as positive. The degree of overlap between the predicted and actual tumor regions in the image is quantified by DSC. A high DSC implies good alignment between the predicted tumor region by the model and the actual tumor region, whereas a low DSC suggests poor alignment. MCC would indicate the overall relationship between the model’s predictions and the actual tumor or non-tumor labels based on the samples’ histological characteristics.

13 $$\begin{aligned} MCC = \frac{TP*TN-FP*FN}{\sqrt{(TP+FN)(TP+FP)(TN+FN)(TN+FP)}} \end{aligned}$$

We separated the dataset into training, validation, and testing categories since a DL model’s training and testing phases are critical. Specific hyperparameters, such as learning rate, batch size, and the number of training epochs, should be carefully selected for the training phase. Table 4 displays the values of the YOLOV5-RESNET50 model’s hyperparameter optimization trials on the dataset.

**Table 4:** The specific hyper parameter of the proposed model.

Component	Hyperparameter	Value
*ResNet50 backbone*	Learning rate	0.0001
	Momentum	0.84
	Weight decay	0.00035
*Modified YOLOv5s backbone*	CSP module modifications	Yes
	Learning rate	0.0001
	Momentum	0.84
	Weight decay	0.00035
*Neck layer (YOLOv5)*	Learning rate	0.001
	Momentum	0.9
	Weight decay	0.0001
*XGBoost classifier*	Learning rate	0.1
	n_estimators	70
	Max_depth	6
	Gamma	0.1
*Whole model*	Learning rate scheduler	CosineAnnealingLR
	Loss function	Similar to YOLOv5
	Optimizer	Adam
	Epochs	200
	Batch Size	32

Choosing the best parameters and values for a DL model is crucial. During this process, the model achieves the best accuracy on the hyperparameters shown in Table 4. The suggested model based on these hyperparameters values is then validated using the validation dataset. The performance metrics were recorded for each epoch of training and validation.

Python 3.9 and Google Colab were used to develop the proposed system. TensorFlow 2.4 was used as the main processing framework to implement this work. Besides, the OpenCV library is used for the preprocessing stage as it is an open-source Python library. Our studies were carried out on a core i7/4.5 GHz computer. It had 16 GB of RAM and a 4GB VRAM NVIDIA card.

### Results

**Figure 6:**
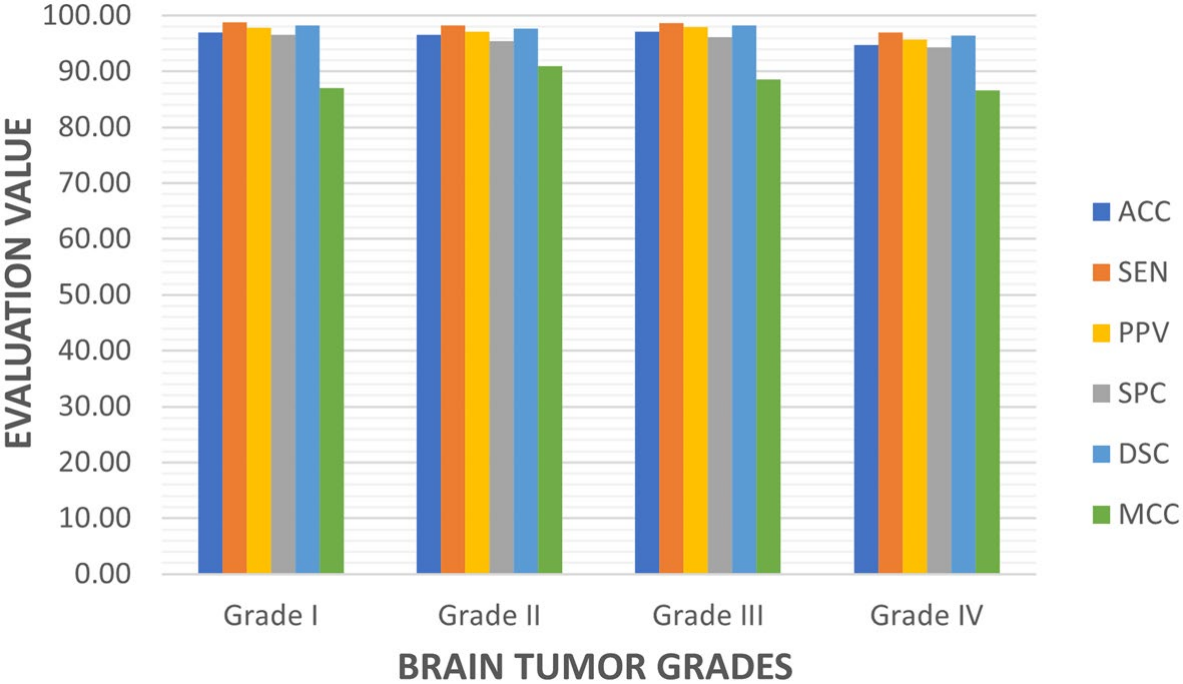
Model evaluation per class.

The suggested model’s performance on previously unseen data has been thoroughly assessed using test data. Fig. 6 shows the model’s performance on the test dataset. With ACC equals to 97.2%, PPV equals to 96.3%, and DSC equals to 97.0% , the model performs well within the first three grades, but grade IV identification is remarkably well. SEN and SPC are also beneficial to all classes. Regarding performance, the ROC curve for all classes is shown in Fig. 5. Figure 6 depicts the use of the four classifiers on a $256 \times 256$ patch size. The ROC curve has been presented in this figure, confirming that the suggested model has the ROC curve for four classes in contrast to the other classifiers. Table 5 shows the classification results of different models trained on $256 \times 256$ patch size for each cross-validation in 5 folds cross-validation. The findings reveal that the suggested model has the highest average indexes (across all four classes). The Model evaluation of different four grades for glioma brain tumor using differnet metrics like ACC,SEN,F1-score,etc is shown in Table 6

**Table 5:** The comparison of the suggested model’s results (%) and some recent CNN techniques.

	ACC	SEN	SPC	DSC	MCC	PPV
InceptionV3	92.2	94.4	88.8	93.6	83.6	92.9
EfficientNet	94.1	96.6	87.9	95.9	85.6	95.2
VGG19	93.7	96.4	88.2	95.4	85.6	94.4
Proposed model	97.2	97.7	94.9	97.0	92.8	96.3

**Table 6:** The model evaluation of different grades for glioma brain tumor.

	ACC (%)	SEN (%)	SPC (%)	PPV (%)	F-score (%)	MCC(%)
Grade I	97.05	98.74	96.60	97.87	98.30	87.05
Grade II	96.55	98.20	95.43	97.17	97.68	90.92
Grade III	97.08	98.67	96.20	97.89	98.28	88.51
Gradde IV	94.69	96.99	94.38	95.73	96.35	86.62

### Discussion

This study presents a new hybrid deep learning model on histopathological images from TCGA dataset for the classification of glioma brain tumors. The suggested model integrates the best aspects of three well-known architectures: XGBoost for robust classification, YOLOv5 for feature selection and object detection, and ResNet50 for feature extraction. In order to decrease redundancy and increase efficiency in the feature extraction process, this study also modifies the YOLOv5 backbone by eliminating the alternate branches of the conv2dx1 section.

Histopathology images have traditionally been utilized in clinical settings to classify tumors. However, there are specific difficulties in processing histopathologic images. First, the size of the WSI varies significantly within the dataset. Second, choosing an RoI is quite difficult. The quality of the chosen RoI influences the ultimate tumor classification. Finally, RoI selection in WSI is quite computationally demanding for this operation.

We achieved good accuracy and robustness in glioma grade classification by utilizing a hybrid YOLOv5 and ResNet50 model for feature extraction, followed by a gradient boosting classifier. To extract high-level semantic characteristics and low-level object features essential for tumor grading, the hybrid model can take advantage of the strengths of both the ResNet50 and YOLOv5 models. After collecting features from the hybrid ResNet50 and YOLOv5 model, the XGBoost classifier is used to categorize brain tumor grades, which is an efficient method for precise and trustworthy tumor grading. Our model, which was a customized integration of modified YOLOv5 backbone and a ResNet50, was optimized and fine-tuned in terms of the number of parameters. Consequently, a model configuration with approximately 28.16 million parameters was produced.On a machine with the NVIDIA GeForce GTX and Intel(R) Core (TM) i7/4.5 GHz specifications, the model’s processing time was measured. An image’s processing takes about 30 milliseconds on average.

We compare the suggested hybrid model to various approaches that have been investigated in the literature that use the WSI from the TCGA. Sumi et al.^23^ achieved 95.6% for ACC. Yonekura et al.^24^ achieved 96.5% for GMB classification. Kurc et al.^63^ presented three classification techniques to group adult diffuse glioma patients into oligodendroglioma and astrocytoma classes. They obtained 75.0%, 80.0%, and 90.0% for ACC using a weighted average-based classification method. Dropout enables ensemble learning for multi-scale image classification and DenseNet-161 network for classifying low-grade gliomas. To further improve the evaluation robustness of our model, we have applied k-fold cross-validation with 10 folds. Using this method, the data is split into ten distinct folds, each of which is used at different stages for training and validation. By doing this, it is made sure the model is tested on a variety of data subsets, which reduces the possibility of overfitting and gives a more complete picture of the model’s generalizability.

**Figure 7:**
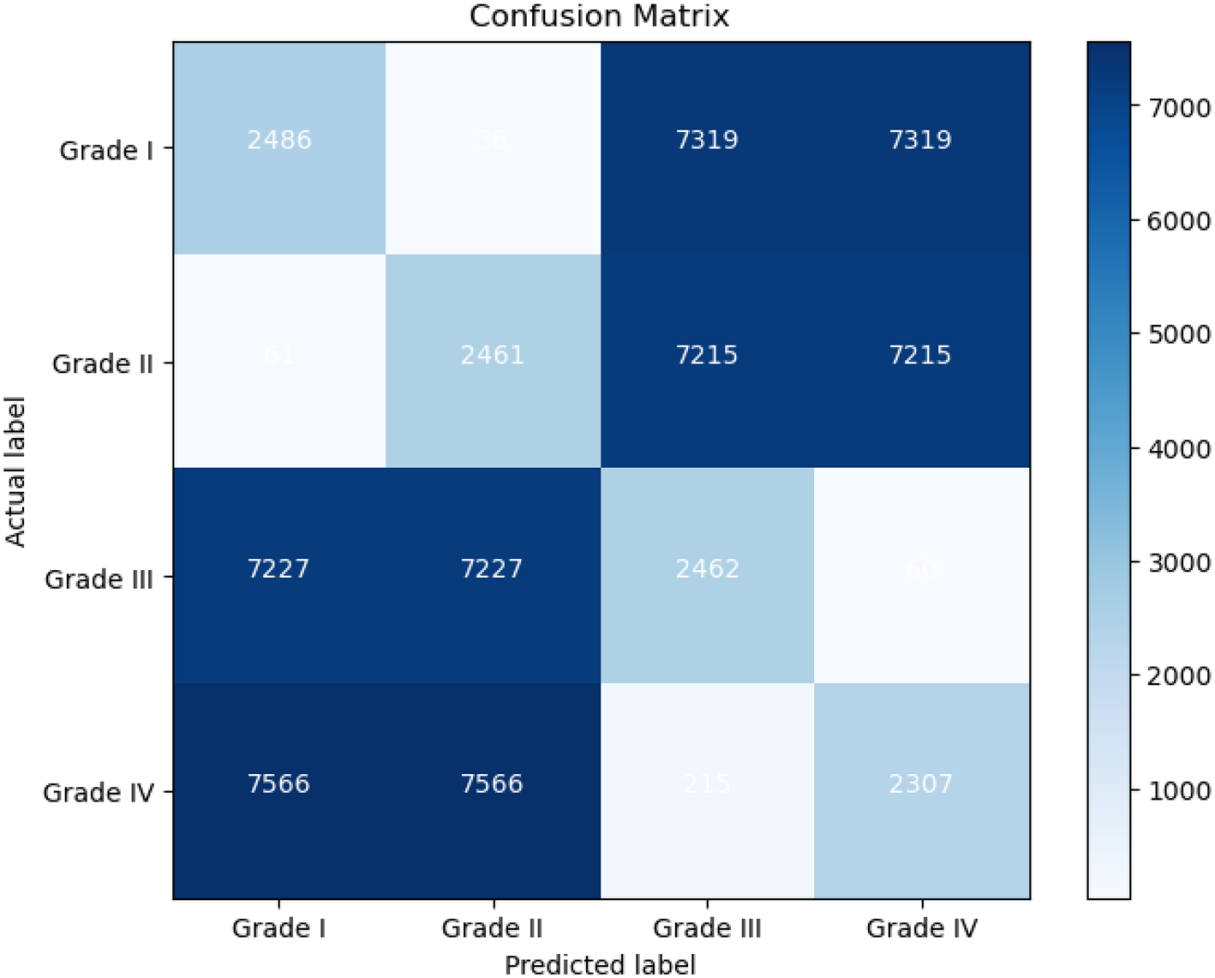
The confusion matrix for the model’s performance.

**Figure 8:**
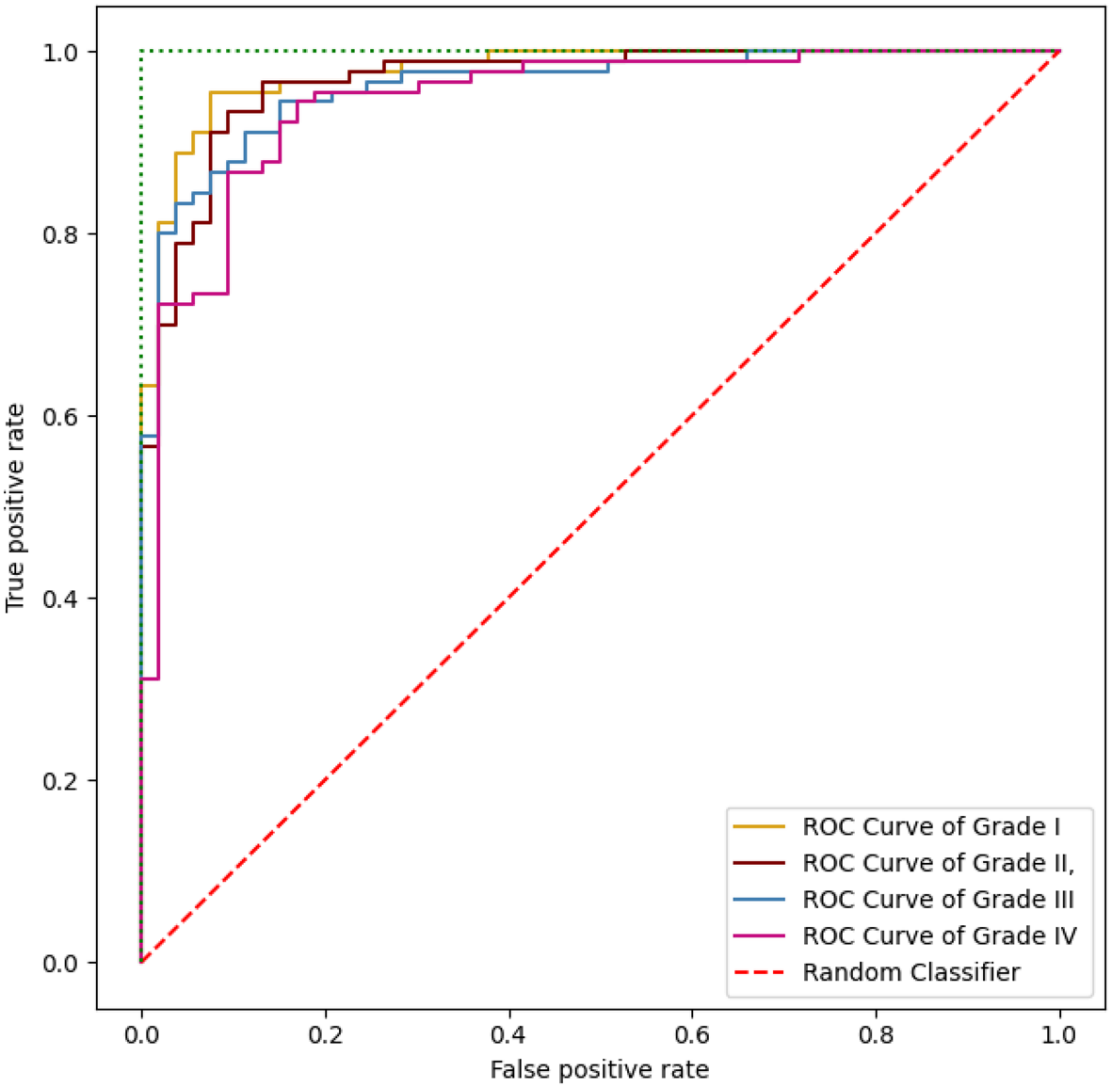
The ROC curve for four grades output.

Compared to the state-of-the-art models, the proposed system produced the best results. Our suggested system outperforms the VGG19, ResNet50, Inception V3, and MobileNetv2 models. We discovered that when we altered the standard ResNet50 by adjusting the hyperparameters and layers, the results needed to be raised to satisfy the glioma grades’ satisfying expectations. Our suggested approach, which combines YOLOv5 with ResNet50, performs better than the other method. This implies that the proposed method is more effective in achieving the desired result. We investigated using H &E-stained brain cancer histopathology pictures as input for DCNN-based glioma classification. We compared the performance of DCNN algorithms using the publicly accessible TCGA dataset. Our suggested model, a hybrid method for identifying and categorizing brain tumors, was developed using a set of training samples from the TCGA dataset. This model is intended to provide a comprehensive approach for precisely classifying and detecting various grades of brain cancer. In the testing phase, the patch classification accuracy using YOLOv5+ResNet50 was 97.2%. A confusion matrix was used to evaluate the model’s performance as shown in Fig. 7. We evaluated the performance of the proposed classification by comparing its results with different state-of-the-art grade classification methods. The result of the comparison with state-of-the-art techniques is shown in Table 7. The results show that the proposed model, which is based on the hybrid of Yolov5 and Resnet50, performs better than other state-of-the-art methods (Fig. 8).

**Figure 9:**
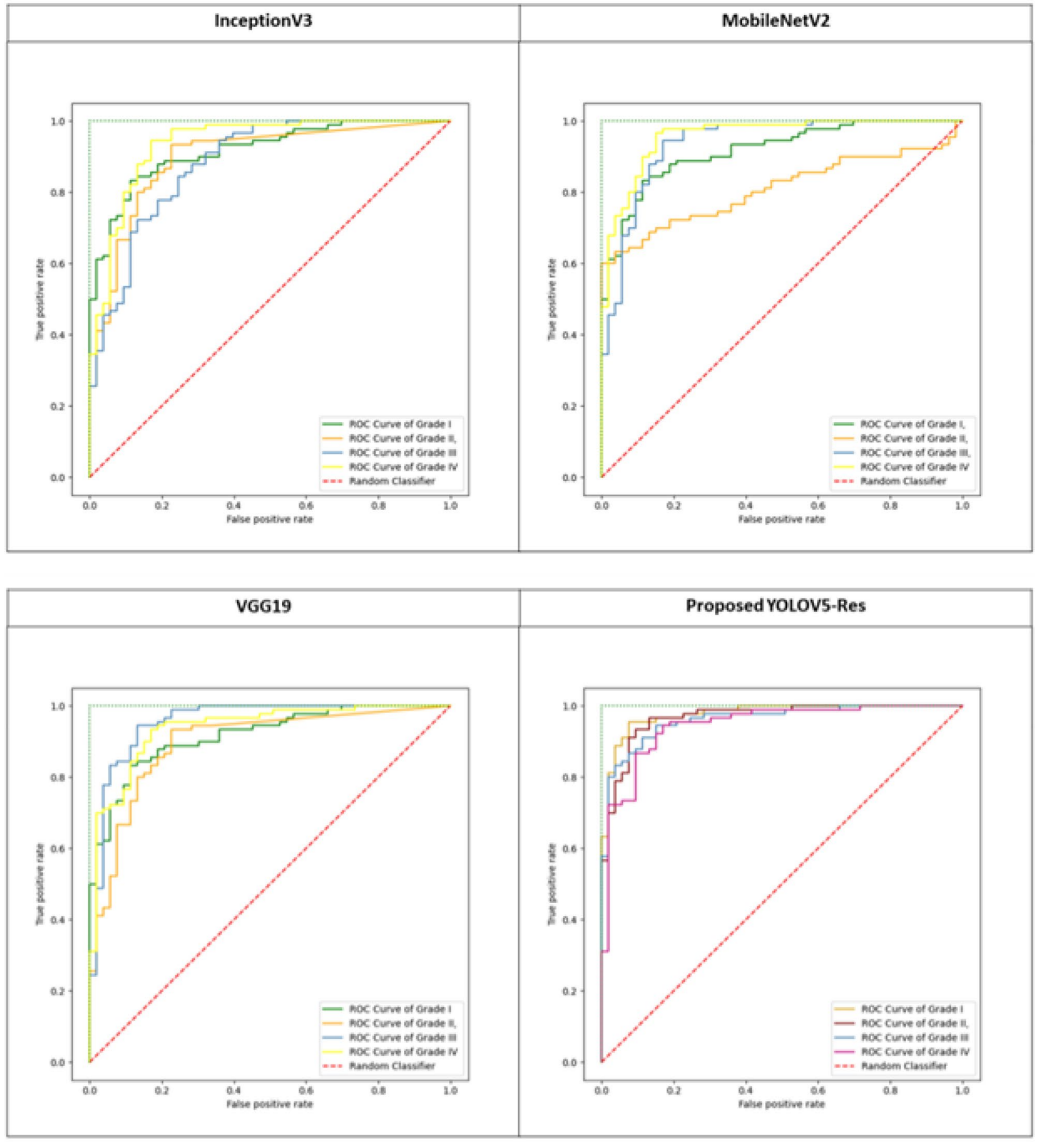
The ROC curve for four classifiers output on patches $256\times 256$: Inception V3, MobileNet V2, VGG19, Proposed model.

When we compared YOLOv5’s original and the proposed model, we found that the proposed model performed better. Figure 6 shows the proposed model’s results, which include 97.7% SEN, 94.9% SPC, 96.3% PPV, 97.2% ACC, and 92.8% MCC value. The original YOLOv5, in contrast, obtained 92% SEN, 91.6% PPV, 91.8% ACC, and 85.4% MCC value. We further evaluated the performance of three models (InceptionV3, EfficientNet, and VGG19) for identifying brain tumors and discovered that the enhanced YOLOv5s model outperformed these models regarding MCC and ACC by 92.8% and 97.2%, respectively. As demonstrated in Fig. 9, our suggested model performs better than InceptionV3, MobileNetV2, and VGG19 in classifying and diagnosing brain tumors. The brain tumor identification and classification outcomes using a proposed network are shown in Fig. 8.

**Table 7:** The comparison between the proposed system and state-of-the-art techniques.

	Method	ACC(%)
Yonekura et al. ^24^	Applied Deep CNN. The approach comprises of 7 convolution, 8 ReLU, 6 pooling, 1 softmax layers	96.5
Sumi et al.^23^	The InceptionResNetV2 is used to extract features	95.0
Pei et al.^44^	Based on cellularity and molecular characteristics and applied DNN classifier	93.8
Im et al. ^49^	Using the ResNet50V2 model	87.2
Mohan ^48^	A feature combination of PF+FLBP+GLCM+GABOR with linear SVM	93.5
The proposed method	Yolov5 integrated with resnet50 for feature extraction and applied XGboost for classification	97.2

Deep learning has been investigated in several studies for brain tumor classification. The propsed model has several benefits over these methods:

- *Enhanced extraction of features:* When YOLOv5 and ResNet50 are used together, the resulting model offers a larger feature set than when either model is used alone.
- *Increased effectiveness:* Removing unnecessary branches keeps performance at a high level while increasing effectiveness.
- *Flexibility and interpretability:* XGBoost facilitates fine-tuning and offers insights into the significance of features, allowing for a better understanding and possible model improvement

The efficacy of the hybrid architecture and the suggested modifications is demonstrated by the proposed model, which achieves notable performance gains on several metrics over individual baseline models and pre-defined CNNs. When compared to other models, the suggested model has the best accuracy in classifying GBM, demonstrating its superior capacity to distinguish between tumor and non-tumor regions. High sensitivity and specificity are displayed by the model, indicating that it has good capabilities for grading the brain tumor. The model demonstrates its generalizability to previously unseen data by exhibiting strong performance across a variety of evaluation metrics.

## Conclusion and future work

It is critical to create non-invasive, inexpensive, and efficient technology for diagnosing and grading gliomas since brain tumors are a common, serious condition with a poor prognosis. In impoverished healthcare systems, a DL framework can be an invaluable replacement for conventional tools, particularly for early preventative treatment. Our research intends to develop a system that can automatically categorize brain tumors using a hybrid model built on YOLOv5 and ResNet50. The modified approach, which incorporates ResNet into the feature extraction of the YOLOv5 framework, is used by our hybrid network to successfully identify brain tumors from histopathological images. However, our method is still unable to identify tumors with atypical forms. Future improvements will improve ease and allow the network to detect tumors of all sorts and sizes. Using this method, we hope to contribute new ideas and focus on classifying different cancers. The method may also be more broadly applicable to a wider variety of clinical situations if additional imaging modalities, such as MRI or CT scans, are investigated. The suggested method’s clinical applicability and effectiveness may also be supported by conducting a clinical validation study to evaluate its performance on a bigger and more varied dataset. Future research into different network architectures, such as DenseNet or EfficientNet, may be useful to see whether they can accomplish the task of classifying brain tumors more effectively.

## Data Availability

The datasets used during the current study available in the Cancer Genome Atlas repository (https://portal.gdc.cancer.gov/) at the TCGA-LGG and TCGA-GBM projects.
